# Chromosomal Localization and Diversity Analysis of 5S and 18S Ribosomal DNA in 13 Species from the Genus *Ipomoea*

**DOI:** 10.3390/genes15101340

**Published:** 2024-10-19

**Authors:** Jingyu Wu, Tao Lang, Cong Zhang, Fan Yang, Feiyang Yang, Huijuan Qu, Zhigang Pu, Junyan Feng

**Affiliations:** 1 Biotechnology and Nuclear Technology Research Institute, Sichuan Academy of Agricultural Sciences, Chengdu 610066, China; 2 Sichuan Academy of Agricultural Sciences, Chengdu 610066, China

**Keywords:** 5S rDNA, 18S rDNA, fluorescence in situ hybridization, phylogenetic analysis, *Ipomoea* genus

## Abstract

**Background:** Sweet potato (*Ipomoea batatas* (L.) Lam.), a key global root crop, faces challenges due to its narrow genetic background. This issue can be addressed by utilizing the diverse genetic resources of sweet potato’s wild relatives, which are invaluable for its genetic improvement. **Methods:** The morphological differences in leaves, stems, and roots among 13 *Ipomoea* species were observed and compared. Chromosome numbers were determined by examining metaphase cells from root tips. Fluorescence in situ hybridization (FISH) was used to identify the number of 5S and 18S rDNA sites in these species. PCR amplification was performed for both 5S and 18S rDNA, and phylogenetic relationships among the species were analyzed based on the sequences of 18S rDNA. **Results:** Three species were found to have enlarged roots among the 13 *Ipomoea* species. Chromosome analysis revealed that *I. batatas* had 90 chromosomes, *Ipomoea pes-tigridis* had 28 chromosomes, while the remaining species possessed 30 chromosomes. Detection of rDNA sites in the 13 species showed two distinct 5S rDNA site patterns and six 18S rDNA site patterns in the 12 diploid species. These rDNA sites occurred in pairs, except for the seven 18S rDNA sites observed in *Ipomoea digitata*. PCR amplification of 5S rDNA identified four distinct patterns, while 18S rDNA showed only a single pattern across the species. Phylogenetic analysis divided the 13 species into two primary clades, with the closest relationships found between *I. batatas* and *Ipomoea trifida*, as well as between *Ipomoea platensis* and *I. digitata*. **Conclusions:** These results enhance our understanding of the diversity among *Ipomoea* species and provide valuable insights for breeders using these species to generate improved varieties.

## 1. Introduction

Sweet potato (*Ipomoea batatas* (L.) Lam.), which is one of the most important root crops, is cultivated worldwide because of its high starch content and abundance of vitamins and other nutrients. It is grown as a source of food, feed, fuel, and starch [1]. Sweet potato varieties that are resistant to diseases and pests and have high yields and nutritional value are crucial for satisfying the growing demand for high-quality food and industrial raw materials [2]. However, the narrow genetic background of sweet potato results in low genetic diversity, thereby hindering varietal improvement [3]. Thus, wild relatives of sweet potato are important genetic resources for enhancing sweet potato cultivars [1]. Accordingly, clarifying the relationships between sweet potato and its wild relatives is important for optimizing the genetic improvement of sweet potato through breeding.

Ribosomal DNA (rDNA) is a highly conserved genomic segment in eukaryotes that is widely used for chromosomal localization and phylogenetic analyses. It consists of 45S and 5S rDNA, with 45S rDNA comprising repeating units of 18S, 5.8S, and 26S rDNA, as well as tandem arrays of transcribed and non-transcribed spacer regions [4]. The number and distribution of rDNA have been analyzed in many species [5]. In the genus *Ipomoea*, 5S and 18S rDNA have been used to provide evidence of the origin of sweet potato [6]. Additionally, 5S rDNA has been used to analyze the karyotypes of *I. batatas* and its wild relatives [7].

Over the past decade, oligonucleotide fluorescence in situ hybridization (oligo-FISH) has become an important technique for analyzing chromosomes [8]. In terms of its utility for cell biology and genetics research, oligo-FISH can accurately locate DNA sequences on chromosomes and chromatin [9], but it is also widely used to identify chromosomes [10] and analyze karyotypes [11] as well as phylogenetic relationships [12,13] among plants and microbes. Furthermore, using rDNA oligonucleotide probes to visualize rDNA chromosomal sites can clarify the variability in 5S and 18S rDNA.

Plastome sequences, ITS (internal transcribed spacer), and rDNA sequences have often been used as reliable molecular evidence of phylogenetic relationships. For example, Chen et al. elucidated the phylogenetic relationships of 40 species from the family Convolvulaceae using complete plastome sequences [14]. The analysis of ITS sequences in *I. batatas* and its wild relatives by Chen et al. and Xu et al. clarified the genetic relationships among these species [15,16]. 18S rDNA sequences and 5S rDNA molecular markers have commonly been employed in phylogenetic analyses [17]. However, there are relatively few reports describing phylogenetic relationships among *Ipomoea* species determined on the basis of 18S rDNA sequences and 5S rDNA molecular markers.

In this study, we examined 13 species from the genus *Ipomoea* in terms of their morphological characteristics, number and distribution of 18S and 5S rDNA sites, and polymorphisms among 18S rDNA sequences and 5S rDNA molecular markers. This study’s results will be useful for exploiting these species for the genetic improvement of sweet potato.

## 2. Materials and Methods

### 2.1. Plant Materials

The seeds of 12 *Ipomoea* species, including *Ipomoea muelleri*, *Ipomoea murucoides*, *Ipomoea trifida*, *Ipomoea triloba*, *Ipomoea nil*, *Ipomoea setosa*, *Ipomoea platensis*, *Ipomoea quamoclit*, *Ipomoea obscura*, *Ipomoea pes-tigridis*, *Ipomoea pes-caprae*, and *Ipomoea digitata*, and seedlings of *I. batatas* were used in this study. All germplasms were supplied by the Biotechnology and Nuclear Technology Research Institute of Sichuan Academy of Agricultural Sciences, China. The sweet potato cultivar Xushu 18 was bred by the Xuzhou Institute of Agricultural Sciences, China. All the seeds were placed in a moist box. After germination, three well-sprouted seeds from each species were selected and transferred into pots filled with soil. The pots were placed in a greenhouse in Chengdu, China. The greenhouse was maintained at 28 °C with 70% relative humidity. Plants were exposed to light from 8:00 a.m. to 10:00 p.m. The examination of phenotypic traits for all species was based on *Descriptors for Sweet Potato* [18].

### 2.2. Chromosome Preparation

Two months after planting, root tips were collected and treated using a published method [19]. Briefly, all root tips were exposed to nitrous oxide for 2 h to inhibit spindle formation, thereby allowing for the observation of more metaphase cells. Then, they were placed in 90% acetic acid for 10 min to fix the cells and stored in 70% ethanol solution. After being thoroughly washed using ddH_2_O, the root tips were placed in a solution comprising 1% pectinase and 2% cellulase (Yakult Pharmaceutical Industry Co., Ltd., Tokyo, Japan) and incubated in a 37 °C water bath for 1 h to digest the cell walls. Following this, the root tips were washed using 70% ethanol and squashed in acetic acid. Finally, 10 μL of the solution was dropped onto each slide and air-dried in a moist box for at least 5 min.

### 2.3. Fluorescence In Situ Hybridization

18S rDNA probe multiplexes were developed and 5S rDNA probes were synthesized as described by Yu et al. [20]. The sequences of the 18S rDNA probe multiplexes and 5S rDNA probes are listed in Table 1.

A droplet of a solution comprising both 18S and 5S rDNA probes was placed on individual slides, which were then covered for 1 h hybridization at 42 °C. After removing the cover glass using 2× SSC (saline–sodium citrate), the samples were air-dried and stained with 4′,6-diamidino-2-phenylindole. FISH images were captured using a Leica DM2500 microscope (Leica Microsystems, Wetzlar, Germany). The resulting photos were processed using Adobe Photoshop 2020 (Adobe Systems Software Ltd., Dublin, Ireland). At least 10 metaphase plates were examined for each species.

### 2.4. DNA Extraction and PCR Amplification of 5S and 18S rDNA

DNA was extracted according to the CTAB method [21]. The primers used for the PCR amplification of 18S rDNA were 18S-forward (5′-CAACCTGGTTGATCCTGCCAGT-3′) and 18S-reverse (5′-CTGATCCTTCTGCAGGTTCACCTAC-3′) [22], whereas 5S-forward (5′-GGATCCCATCAGAACTCC-3′) and 5S-reverse (5′-GGTGCTTTAGTGCTGGTAT-3′) were the primers used for the PCR amplification of 5S rDNA [23]. Both PCR amplifications were performed in 20 µL reaction mixtures containing 10 μL 2× Taq PCR Master Mix (Bio Basic Inc., New York, NY, USA), 2 µL DNA template, 0.2 μL forward and reverse primers (10 μM), and 7.6 μL ddH_2_O. The PCR cycling conditions were as follows: initial denaturation at 94 °C for 5 min; 35 cycles of denaturation at 94 °C for 30 s, primer annealing at 58 °C for 30 s, and primer extension at 72 °C for 2 min and 20 s; and a final extension at 72 °C for 7 min. Amplified products were visualized via 1.5% (*w*/*v*) agarose gel electrophoresis.

### 2.5. 18S rDNA Sequence Analysis

Amplified fragments were purified from the agarose gel using Universal DNA Purification Kit (Tiangen, Beijing, China). Then, the purification products were sequenced using Sanger sequencing method by Tsingke Biotechnology Co., Ltd. (Beijing, China). DNA sequences were aligned using the MUSCLE algorithm, which was widely used in sequences analysis for its high accuracy and reliability. A phylogenetic tree was constructed according to the neighbor-joining method with 1000 bootstrap replicates using MEGA 11. tvBOT (https://www.chiplot.online/circleTree.html, accessed on 31 August 2023) [24], a tool to customize the layout and font style, was employed to visualize the phylogenetic tree. Genetic distances were calculated on the basis of the Kimura 2-parameter model.

## 3. Results

### 3.1. Phenotypic Analysis of 13 Ipomoea Species

**Figure 1:**
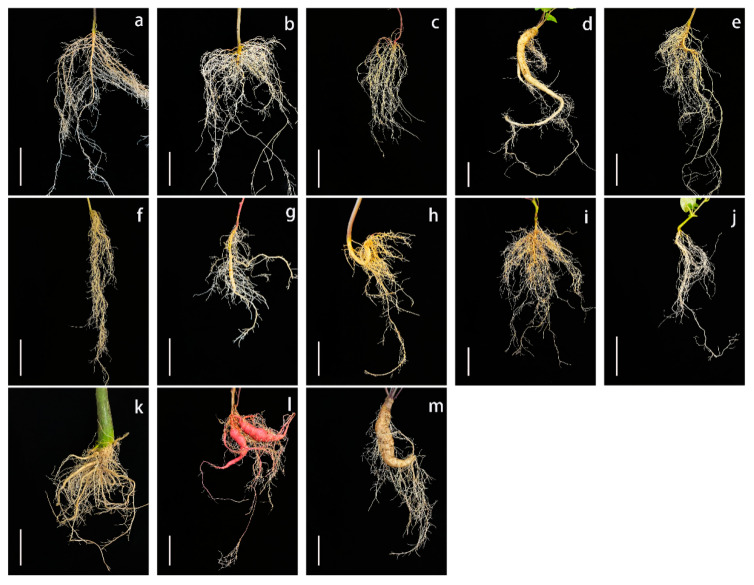
Comparison of root morphology among 13 species of the genus *Ipomoea*. (**a**): *Ipomoea triloba*; (**b**): *Ipomoea nil*; (**c**): *Ipomoea quamoclit*; (**d**): *Ipomoea platensis*; (**e**): *Ipomoea pes-tigridis*; (**f**): *Ipomoea pes-caprae*; (**g**): *Ipomoea trifida*; (**h**): *Ipomoea setosa*; (**i**): *Ipomoea obscura*; (**j**): *Ipomoea muelleri*; (**k**): *Ipomoea murucoides*; (**l**): *Ipomoea batatas*; (**m**): *Ipomoea digitata*. Scale bars, 5 cm.

The root, stem, and leaf phenotypes of 13 *Ipomoea* species were analyzed (Figure 1, Figures S1 and S2).

The leaves of all species varied in terms of shape, size, and color. Notably, leaves with downy hair were detected exclusively on *I. nil* and *I. pes-tigridis* plants. Pinnately lobed leaves were detected only on *I. quamoclit* plants. *I. nil*, *I. pes-tigridis* and *I. setosa* all had palmately lobed leaves. However, *I. nil* and *I. setosa* had moderate lobed leaves, while *I. pes-tigridis* had deep lobed leaves. Interestingly, *I. platensis* and *I. digitata* had both very deep lobed and very slight lobed leaves. Although all species had green leaves, they differed in leaf vein and petiole colors. Specifically, the leaf veins of *I*. *trifida*, *I. setosa*, *I. pes-caprae*, and *I. batatas* exhibited anthocyanin pigmentation. Additionally, the petioles of these four species, along with *I. triloba*, *I. nil*, and *I. obscura*, also showed anthocyanin pigmentation.

The stems differed in terms of shape and thickness. *I. murucoides* had erect stems, but all other species had twining stems. Furthermore, downy hair was detected on the stems of *I. nil*, *I. setosa*, and *I. pes-tigridis*. In addition to being the only species with an erect stem, *I. murucoides* also had the thickest stem (approximately 14 mm in diameter). *I*. *muelleri* and *I. obscura* had the thinnest stems (approximately 1 mm in diameter). For all other species, stem thickness ranged from 2.5 to 5 mm.

There were significant root morphological differences. The roots of three species clearly expanded, whereas the roots of the other ten species did not. Both *I. platensis* and *I. digitata* had only one long irregular storage root, while *I. batatas* produced multiple long elliptic storage roots. Moreover, the storage roots of *I. platensis* and *I. digitata* were both developed from the swelling of the primary root. In contrast, the storage roots of *I. batatas* were developed from multiple lateral roots. Among the examined species, only *I. batatas* had red skin on its storage roots.

### 3.2. Number and Length of Chromosomes

**Figure 2:**
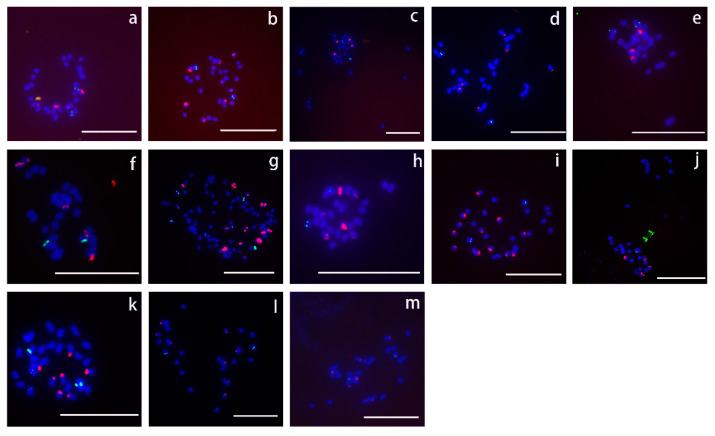
Chromosomes and rDNA sites in 13 species of the genus *Ipomoea*. 5S rDNA is green, and 18S rDNA is red. (**a**): *Ipomoea obscura*; (**b**): *Ipomoea quamoclit*; (**c**): *Ipomoea setosa*; (**d**): *Ipomoea trifida*; (**e**): *Ipomoea murucoides*; (**f**): *Ipomoea pes-caprae*; (**g**): *Ipomoea batatas*; (**h**): *Ipomoea pes-tigridis*; (**i**): *Ipomoea nil*; (**j**): *Ipomoea muelleri*; (**k**): *Ipomoea digitata*; (**l**): *Ipomoea platensis*; (**m**): *Ipomoea triloba*. Scale bars, 10 μm.

With the exception of *I. batatas*, which was revealed to be a hexaploid with 90 chromosomes, the remaining species were diploids with 30 chromosomes, except for *I. pes-tigridis*, which had 28 chromosomes (Table 2, Figure 2).

A comparison of chromosome lengths (Table 3) indicated *I. batatas* had the shortest chromosomes (average length of 0.52 μm; the longest chromosome was 2.795 times longer than the shortest chromosome). *I. digitata* had the longest chromosomes (average length of 1.07 μm). The smallest difference in chromosome length was detected in *I. platensis* (the longest chromosome was only 1.444 times longer than the shortest chromosome).

### 3.3. 5S rDNA FISH Signal Sites and Amplified Fragment Polymorphisms

**Figure 3:**
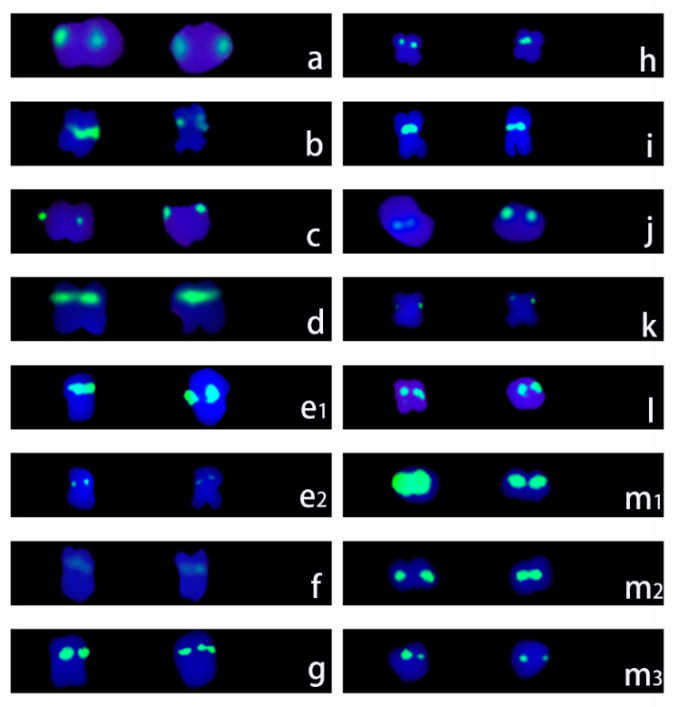
5S rDNA signals in 13 species of the genus *Ipomoea*. (**a**): *Ipomoea obscura*; (**b**): *Ipomoea setosa*; (**c**): *Ipomoea murucoides*; (**d**): *Ipomoea pes-caprae*; (**e_1_**,**e_2_**): *Ipomoea digitata*; (**f**): *Ipomoea triloba*; (**g**): *Ipomoea platensis*; (**h**): *Ipomoea nil*; (**i**): *Ipomoea trifida*; (**j**): *Ipomoea pes-tigridis*; (**k**): *Ipomoea muelleri*; (**l**): *Ipomoea quamoclit*; (**m_1_**,**m_2_**,**m_3_**): *Ipomoea batatas*.

In the metaphase chromosomes of the 13 *Ipomoea* species, 5S rDNA signals were detected in pairs (Figure 3).

There were six and four 5S rDNA signals in *I. batatas* and *I. digitata*, respectively, which was more than the two 5S rDNA signals in the other species. The different signal pairs in *I. batatas* and *I. digitata* differed in terms of intensity. One pair of signals in *I. digitata* was significantly weaker than the other signal pairs. In *I. batatas*, one pair of signals was obviously stronger than the other signal pairs. However, any two signals on homologous chromosomes had essentially the same intensity.

**Figure 4:**
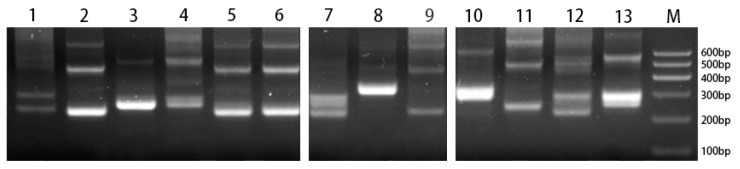
Electrophoresis results of 5S rDNA amplification products in 13 species of the genus *Ipomoea*. 1: *Ipomoea muelleri*; 2: *Ipomoea murucoides*; 3: *Ipomoea trifida*; 4: *Ipomoea triloba*; 5: *Ipomoea nil*; 6: *Ipomoea setosa*; 7: *Ipomoea platensis*; 8: *Ipomoea quamoclit*; 9: *Ipomoea obscura*; 10: *Ipomoea pes-tigridis*; 11: *Ipomoea pes-caprae*; 12: *Ipomoea digitata*; 13: *Ipomoea batatas*; M: Marker.

5S rDNA molecular markers were used to detect polymorphisms in 13 *Ipomoea* species (Figure 4).

There were four patterns of 5S rDNA amplification products. The first pattern, which included two major amplification products (detected as approximately 220 and 450 bp bands), was obtained for *I. murucoides*, *I. nil*, *I. setosa*, and *I. obscura*. The second pattern, which consisted of two major bands at approximately 270 and 520 bp, was detected for *I. trifida*, *I. triloba*, *I. pes-caprae*, and *I. batatas*. The third pattern, which was obtained for *I. muelleri*, *I. platensis*, and *I. digitata*, comprised two major bands (approximately 230 and 290 bp). The final amplification pattern consisted of only one major band (approximately 300 bp) and was detected for *I. quamoclit* and *I. pes-tigridis*.

### 3.4. 18S rDNA Sites and Sequence Polymorphisms

**Figure 5:**
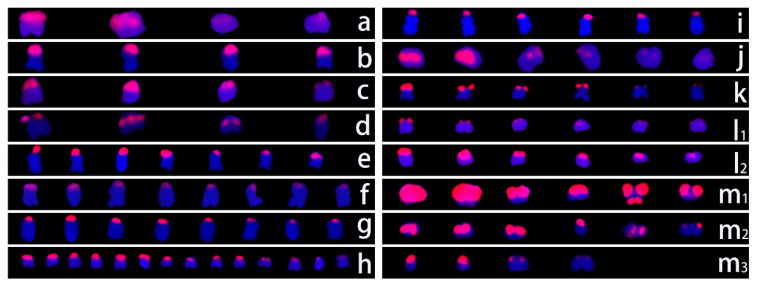
18S rDNA signals in 13 species of the genus *Ipomoea*. (**a**): *Ipomoea obscura*; (**b**): *Ipomoea setosa*; (**c**): *Ipomoea murucoides*; (**d**): *Ipomoea pes-caprae*; (**e**): *Ipomoea digitata*; (**f**): *Ipomoea triloba*; (**g**): *Ipomoea platensis*; (**h**): *Ipomoea nil*; (**i**): *Ipomoea trifida*; (**j**): *Ipomoea pes-tigridis*; (**k**): *Ipomoea muelleri*; (**l_1_**,**l_2_**): *Ipomoea quamoclit*; (**m_1_**,**m_2_**,**m_3_**): *Ipomoea batatas*.

The number of 18S rDNA signals among the 13 selected species could be divided into seven patterns. All 18S rDNA signals were detected as pairs, except in *I. digitata*, which had seven 18S rDNA signals. The only hexaploid species (*I. batatas*) had 16 18S rDNA signals. In the diploid species, four signals were detected in *I. murucoides*, *I. setosa*, *I. obscura*, and *I. pes-caprae*; six signals were detected in *I. muelleri*, *I. trifida*, and *I. pes-tigridis*; and eight signals were detected in *I. triloba* and *I. platensis*. In *I. digitata*, *I. quamoclit*, and *I. nil*, 7, 12, and 14 18S rDNA signals were detected, respectively (Table 2, Figure 5).

All of the 18S rDNA FISH signals in *I. setosa*, *I. triloba*, and *I. trifida* had almost the same intensity, whereas there were obvious differences in the signal intensities in the other 10 species. In addition, the PCR amplification of 18S rDNA generated only one product (approximately 1700 bp) in all 13 species (Figure S3).

### 3.5. Polymorphisms in 18S rDNA Sequences

To further analyze 18S rDNA polymorphisms, we sequenced the amplification products for all 13 species. According to the results, the 18S rDNA sequence lengths for the 13 species ranged from 1682 bp (*I. murucoides* and *I. pes-caprae*) to 1722 bp (*I. quamoclit*). When all 18S rDNA sequences were included, the average length was 1688 bp. Further aligning and splicing of the 18S rDNA sequences (1908 bp) revealed 652 variable sites and 636 parsimony-informative sites, accounting for 34.2% and 33.3% of the sequences, respectively. There were also 16 singleton sites, accounting for 0.8% of the sequences. Moreover, there was relatively little difference in the GC content among species, with an average of 50.7%.

**Figure 6:**
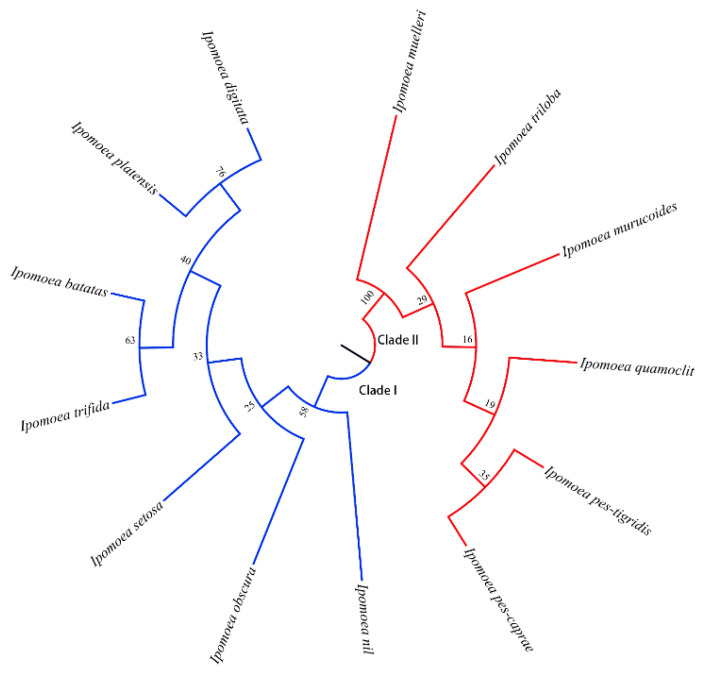
Phylogenetic tree of 13 *Ipomoea* species based on 18S rDNA sequence. The bootstrap analysis was replicated 1000 times. The number shown in each branch indicates the bootstrap value percentage (%).

On the basis of the 18S rDNA sequences in the 13 species, a neighbor-joining phylogenetic tree was constructed (1000 bootstrap replicates) using MEGA 11. The 13 species were divided into two main clades (Figure 6).

Clade I consisted of *I. trifida*, *I. batatas*, *I. platensis*, *I. digitata*, *I. setosa*, *I. obscura*, and *I. nil*, among which *I. trifida* and *I. batatas,* as well as *I. platensis* and *I. digitata,* were clustered together. Clade II included *I. muelleri*, *I. triloba*, *I. murucoides*, *I. quamoclit*, *I. pes-tigridis*, and *I. pes-caprae*, of which *I. pes-tigridis* and *I. pes-caprae* were clustered together.

The 18S rDNA sequences and MEGA 11 were used to calculate genetic distances (Table 4).

The genetic distance between any two species in clade I ranged from 0.000 to 0.003. Notably, the genetic distance between *I. batatas* and *I. trifida*, as well as between *I. platensis* and *I. digitata,* was 0.000. A comparison of the 18S rDNA sequences in *I. batatas* and *I. trifida* detected only three differences. Additionally, the 18S rDNA sequences of *I. platensis* and *I. digitata* were identical. In clade II, the genetic distance between any two species ranged from 0.001 to 0.006. Moreover, the genetic distance between any two species from different main clades ranged from 0.657 to 0.663.

## 4. Discussion

To date, the limited genetic diversity of sweet potato has limited varietal improvement [1,3]. Hence, the introduction of more germplasm resources with substantial genetic diversity is essential for sweet potato breeding. In addition to being genetically diverse, the wild relatives of sweet potato possess desirable characteristics (e.g., disease resistance and high starch content) [25], making them potentially useful for broadening the genetic background of sweet potato [26]. However, there are few reports describing the utility of these wild relatives, except for *I. trifida*, for sweet potato breeding. In this study, we compared the phenotypes of 13 *Ipomoea* species, which revealed considerable differences in the leaves, stems, and roots. For example, *I. murucoides* had a thick and erect stem. Notably, besides sweet potato (*I. batatas*), *I. platensis* and *I. digitata* had expanded roots. These results are relevant to future studies and the application of these germplasm.

Root expansion, which is a key trait of sweet potato, is affected and regulated by many factors [27], including environmental conditions [28], endogenous hormones [29], transcription factors, and genes [30,31,32]. In particular, genes related to the synthesis and metabolism of hormones, lignin, and starch play an important role [33,34]. In recent years, the hypothesis that the formation of sweet potato storage roots may be triggered by *Agrobacterium rhizogenes* derived from species related to sweet potato via hybridization or infection has been proposed [35,36]. Because of the polyploidy and high heterozygosity of sweet potato, the key genes and molecular mechanisms regulating root expansion have not been fully characterized. The two diploid (2*n* = 30) *Ipomoea* species with expanded roots in this study may be useful materials for research on sweet potato root expansion.

Considering the basic chromosome number for the genus *Ipomoea* (n = 15), diploid species should contain 2*n* = 30 chromosomes. Earlier research indicated that *I. trifida*, *I. triloba*, *I. setosa*, *I. nil*, *I. pes-caprae*, and *I. obscura* have 30 chromosomes [6,37,38,39], which is consistent with our findings. Previous studies on *I. pes-tigridis* revealed the following number of chromosomes: 2*n* = 2x = 26, 2*n* = 2x = 30, and 2*n* = 4x = 60 [40,41,42]. However, the number of chromosomes reported by Sampathkumar et al. (2*n* = 2x = 28) was in accordance with our results [39]. Similar studies have also found chromosomal aneuploidy in many *Ipomoea* species [13,43,44]. A few studies suggest that chromosomal aneuploidy affects transcript dosage, ultimately leading to phenotypic variations [45]. The effects of the number of chromosomal mutations remain unknown. The variations in the number of *I. pes-tigridis* chromosomes will need to be investigated further.

rDNA, which is highly repetitive and conserved across various species, has been commonly used as a reliable and stable marker for cytological studies. Analyses of the number and distribution of rDNA sites using FISH probes can help elucidate chromosomal behavior. In the present study, we analyzed 18S/5S rDNA distribution patterns in 13 *Ipomoea* species and found that the number of 18S and 5S rDNA sites in *I. trifida*, *I. nil*, and *I. setosa* was consistent with the results of earlier studies [6,46].

Except for *I. digitata*, which had four 5S rDNA sites, all diploid species possessed two 5S rDNA sites, representing exactly one-third of the number of 5S rDNA sites found in hexaploid species. In contrast, there were seven patterns for the number of 18S rDNA signals. Eight and sixteen signals were detected for *I. triloba* and *I. batatas*, respectively. However, Srisuwan et al. reported that *I. triloba* has 6 18S rDNA sites, while sweet potato (*I. batatas*) from various regions differs regarding the number of 18S rDNA sites (e.g., 12, 16, and 18) [6]. The intraspecific variation in the number of 18S rDNA sites, which is common among plants [47,48,49,50], is related to unequal crossing over and transposition events, chromosomal structure fracture and rearrangement, and polyploidization-related process changes to varying degrees [13]. Interestingly, the number of 5S rDNA sites is relatively stable in *I. batatas*. In this study, as well as in some earlier studies [6,13,23], six 5S rDNA sites were detected, with a few exceptions. Moreover, seven 18S rDNA sites (i.e., not paired) were detected in *I. digitata*. The loss of rDNA sites has been reported for *Citrullus* species [51] and sweet potato [13]. This loss is caused by chromosomal deletion, duplication, and unequal exchange [52]. Furthermore, different signal sizes and intensities for 18S and 5S rDNA sites were observed in this study, which is consistent with the findings of other studies [53,54,55]. This diversity is mainly related to differences in the copy number among rDNA sites [13].

In eukaryotes, rDNA sequences are highly conserved. Many studies that amplified rDNA sequences revealed patterns that were similar to those in the current study, in which only one 18S rDNA sequence [22,56] and multiple 5S rDNA sequences [57,58,59] were amplified. These results confirmed that 18S rDNA is more conserved than 5S rDNA. 5S rDNA multigene family members consist of a highly conserved coding sequence (120 bp) and a variable non-transcribed spacer (NTS), forming hundreds to thousands of tandem repeats [60]. The different 5S rDNA amplification patterns are probably due to how freely NTS can mutate [61]. Using the same primers as us, Choi et al. amplified two 5S rDNA sequences in three different sweet potato cultivars (250 and 340 bp) [23].

The origin of sweet potato remains unclear [62], necessitating further research regarding the phylogenetic relationships among its wild relatives. Of the *Ipomoea* species we selected, *I. batatas*, *I. trifida*, and *I. triloba* had similar 5S rDNA amplification patterns. In addition, the genetic distance between *I. trifida* and *I. batatas* was 0.00 according to the 18S rDNA sequences, which was almost consistent with the findings of a previous related study that examined ITS sequences (0.01) [16]. However, *I. triloba* and *I. batatas* were clearly separated in the phylogenetic tree, with a genetic distance of 0.658. According to previous investigations, *I. trifida* [6,62,63] and *I. triloba* [16,64] are both the progenitors of cultivated sweet potato, but our results indicate that *I. trifida* is more likely to be the progenitor of cultivated sweet potato than *I. triloba*.

In our phylogenetic tree, species located on different main clades showed significant genetic distances. However, species within the same clade exhibit extremely close genetic distances. These results demonstrated that the phylogenetic tree construct by 18S rDNA could be used to effectively distinguish species within the *Ipomoea* genus based on sequences accumulated variations during evolution. Interestingly, the three species that produced enlarged roots were all on clade I, while only the species on clade II displayed the fourth amplification pattern of 5S rDNA. However, the number of 18S rDNA sites and the amplification pattern of 5S rDNA on the two main clades both have multiple patterns, indicating that the polymorphism of rDNA site number and amplification results should be related to genome complexity or polyploidy but not to the variation of the sequence itself. Moreover, *I. platensis* and *I. digitata* were closely related to sweet potato, sharing identical 18S rDNA sequences and similar morphological features (e.g., expanded roots). These characteristics further reflect the importance of these species for future theoretical research and genetic improvement of sweet potato.

Considering the size of the genus *Ipomoea*, our study involved relatively few species. Nevertheless, we detected significant variability in the morphology, number of chromosomes, location of 5S/18S rDNA sites, and 18S rDNA sequences among the selected species. These findings have deepened our understanding of the genus *Ipomoea* and serve as useful information for future investigations. To explore and exploit more wild relatives of sweet potato, additional research involving more species is needed.

## 5. Conclusions

In this study, we clarified the phenotypic diversity among thirteen *Ipomoea* species, with three species, including sweet potato, found to produce expanded roots. Except for sweet potato, which was a hexaploid, all other species were diploid. Furthermore, the number of 18S rDNA sites showed more polymorphisms than that of 5S rDNA. All sites were paired, except for the 18S rDNA sites in *I. digitata*. The amplification patterns of 5S rDNA were found to be more variable, while those of 18S rDNA were more conserved. Thirteen *Ipomoea* species were divided into two main clades based on the analysis of 18S rDNA sequences, and greater genetic distances were observed between the clades. Three species with enlarged roots were all on clade I, and the closest relationships were found between sweet potato and *I. trifida*. These results provide comprehensive information regarding the morphological, molecular, and cytological characteristics of 13 *Ipomoea* species. This information should provide breeders with helpful clues regarding how to optimize the use of these germplasms in breeding programs.

## Figures and Tables

**Table 1:** Sequences of 18S rDNA probe multiplexes and 5S rDNA probe used in this study.

Probe	Sequence	Length (bp)
18S-1	TTTGATGGTACCTACTACTCGGATAACCGTAGT	33
18S-2	GGTAGGATAGTGGCCTACCATGGTGGTGACGGGTG	35
18S-3	TCGAGTCTGGTAATTGGAATGAGTACAATCTAA	33
18S-4	AAAGCAAGCCTACGCTCTGTATACATTAGCATGG	34
18S-5	AGATACCGTCCTAGTCTCAACCATAAACGATGCC	34
18S-6	CTCAACACGGGGAAACTTACCAGGTCCAGACATAG	35
18S-7	GGTCTGTGATGCCCTTAGATGTTCTGGGCCGCACG	35
18S-8	TTGTACACACCGCCCGTCGCTCCTACCGATTGAAT	35
5S	TCAGAACTCCGAAGTTAAGCGTGCTTGGGCGAGAGTAGTAC	41

Note: 18S rDNA probe multiplexes were composed of 18S-1 to 18S-8 and were developed in the present study.

**Table 2:** Chromosome and rDNA site numbers of different *Ipomoea* species.

Species	Chromosome Number	Number of rDNA	
		5S rDNA	18S rDNA
*Ipomoea muelleri*	2*n* = 2x = 30	2	6
*Ipomoea murucoides*	2*n* = 2x = 30	2	4
*Ipomoea trifida*	2*n* = 2x = 30	2	6
*Ipomoea triloba*	2*n* = 2x = 30	2	8
*Ipomoea nil*	2*n* = 2x = 30	2	14
*Ipomoea setosa*	2*n* = 2x = 30	2	4
*Ipomoea platensis*	2*n* = 2x = 30	2	8
*Ipomoea quamoclit*	2*n* = 2x = 30	2	12
*Ipomoea obscura*	2*n* = 2x = 30	2	4
*Ipomoea pes-tigridis*	2*n* = 2x = 28	2	6
*Ipomoea pes-caprae*	2*n* = 2x = 30	2	4
*Ipomoea digitata*	2*n* = 2x = 30	4	7
*Ipomoea batatas*	2*n* = 6x = 90	6	16

**Table 3:** Chromosome length of 13 *Ipomoea* species.

Species	Chromosome Length (nm)		
	Maximum	Minimum	Average
*Ipomoea muelleri*	0.965	0.555	0.744
*Ipomoea murucoides*	0.943	0.369	0.707
*Ipomoea trifida*	1.226	0.667	0.955
*Ipomoea triloba*	1.120	0.534	0.871
*Ipomoea nil*	1.025	0.542	0.817
*Ipomoea setosa*	1.185	0.673	0.839
*Ipomoea platensis*	1.188	0.822	0.994
*Ipomoea quamoclit*	0.934	0.500	0.680
*Ipomoea obscura*	0.911	0.562	0.724
*Ipomoea pes-tigridis*	0.847	0.453	0.663
*Ipomoea pes-caprae*	1.072	0.634	0.807
*Ipomoea digitata*	1.448	0.752	1.072
*Ipomoea batatas*	0.917	0.328	0.569

**Table 4:** Genetic distances among 13 *Ipomoea* species.

Species	1	2	3	4	5	6	7	8	9	10	11	12	13
1	0.000												
2	0.003	0.000											
3	0.658	0.658	0.000										
4	0.003	0.001	0.658	0.000									
5	0.657	0.657	0.002	0.657	0.000								
6	0.659	0.659	0.002	0.659	0.003	0.000							
7	0.657	0.657	0.001	0.657	0.003	0.003	0.000						
8	0.003	0.003	0.660	0.002	0.659	0.661	0.659	0.000					
9	0.659	0.659	0.003	0.659	0.003	0.003	0.003	0.661	0.000				
10	0.006	0.004	0.660	0.005	0.660	0.661	0.660	0.005	0.661	0.000			
11	0.003	0.003	0.661	0.004	0.661	0.663	0.661	0.004	0.663	0.005	0.000		
12	0.657	0.657	0.001	0.657	0.003	0.003	0.000	0.659	0.003	0.660	0.661	0.000	
13	0.658	0.658	0.000	0.658	0.002	0.002	0.001	0.660	0.003	0.660	0.661	0.001	0.000

Note: 1: *Ipomoea muelleri*; 2: *Ipomoea murucoides*; 3: *Ipomoea trifida*; 4: *Ipomoea triloba*; 5: *Ipomoea nil*; 6: *Ipomoea setosa*; 7: *Ipomoea platensis*; 8: *Ipomoea quamoclit*; 9: *Ipomoea obscura*; 10: *Ipomoea pes-tigridis*; 11: *Ipomoea pes-caprae*; 12: *Ipomoea digitata*; 13: *Ipomoea batatas*.

## Data Availability

The original contributions presented in the study are included in the article and Supplementary Material, further inquiries can be directed to the corresponding author.

## Supplementary Materials

The following supporting information can be downloaded at https://www.mdpi.com/article/10.3390/genes15101340/s1, Figure S1: Comparison of stem morphology among 13 species of the genus *Ipomoea*. Figure S2: Comparison of leaf morphology among 13 species of the genus *Ipomoea*. Figure S3: Electrophoresis results of 18S rDNA amplification products in 13 species of the genus *Ipomoea*.
