# Supplementary material for: Chromosomal Localization and Diversity Analysis of 5S and 18S Ribosomal DNA in 13 Species from the Genus Ipomoea
Source: Genes (Basel). 2024 Oct 19;15(10):1340. doi: 10.3390/genes15101340 (PMC11508114; doi:10.3390/genes15101340)
Supplement: Supplementary file 1 [file genes-15-01340-s001.zip › genes-3236487-supplementary.pdf]

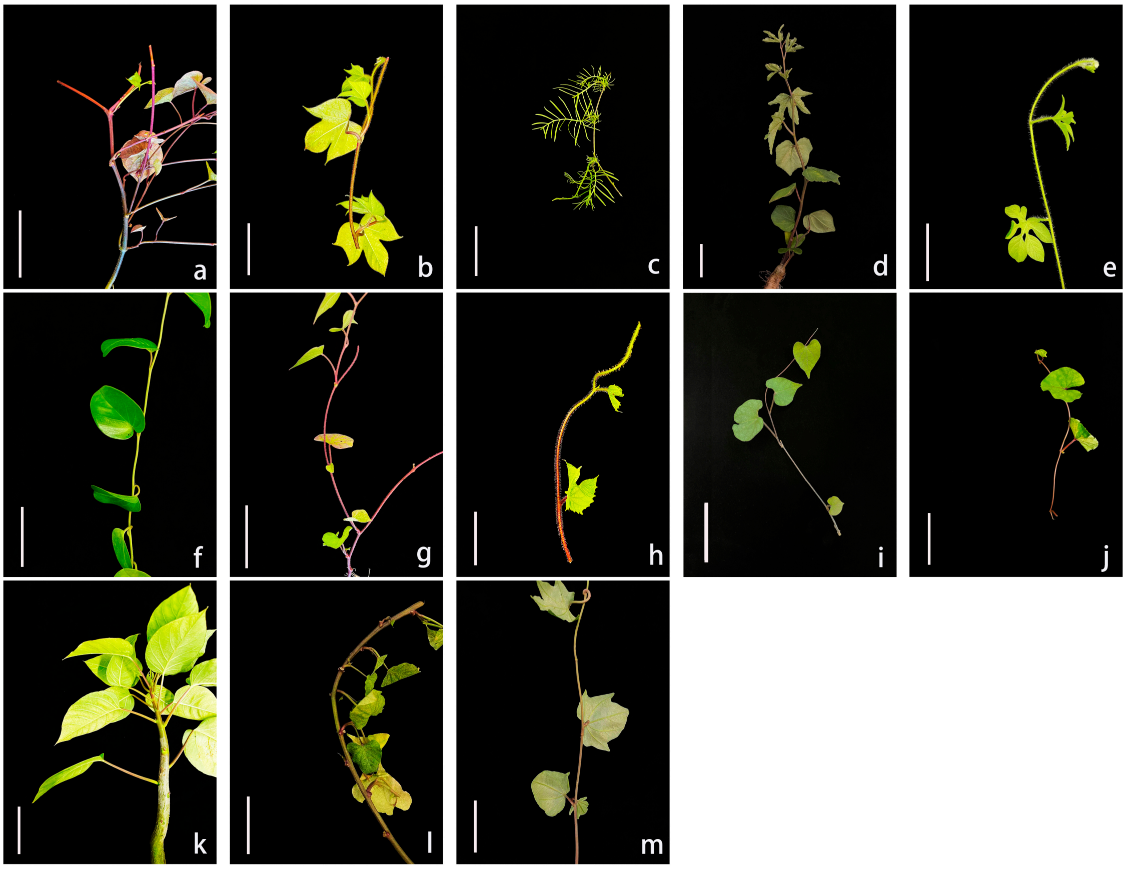

Figure S1. Comparison of stem morphology among 13 species of the genus *Ipomoea*.

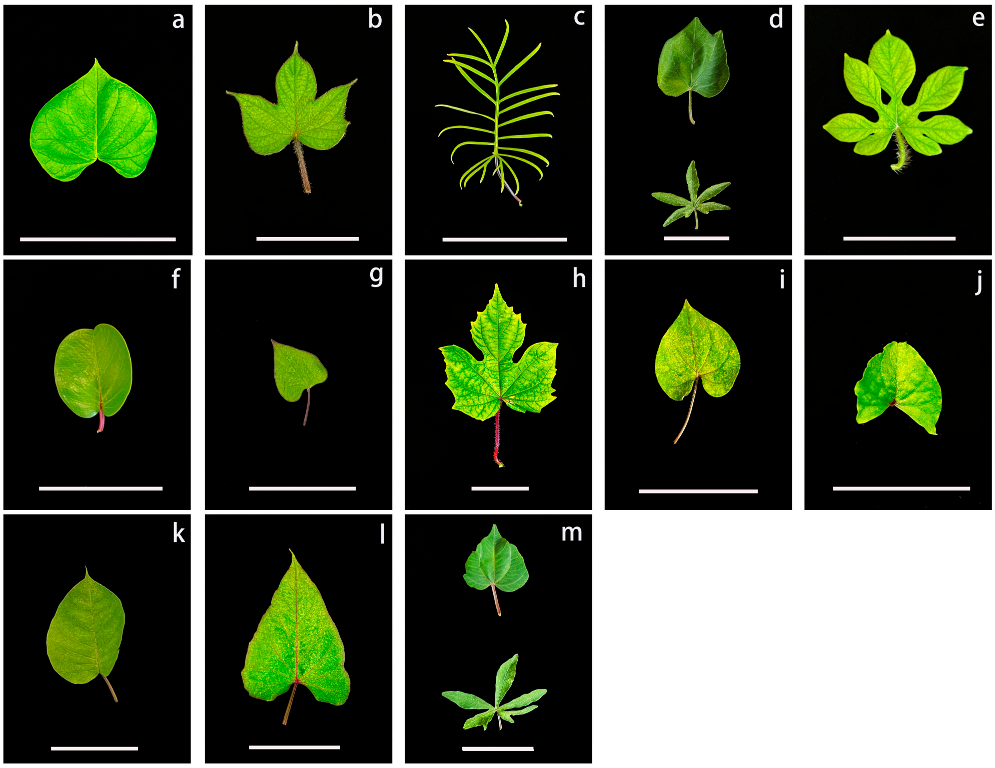

Figure S2. Comparison of leaf morphology among 13 species of the genus *Ipomoea*.

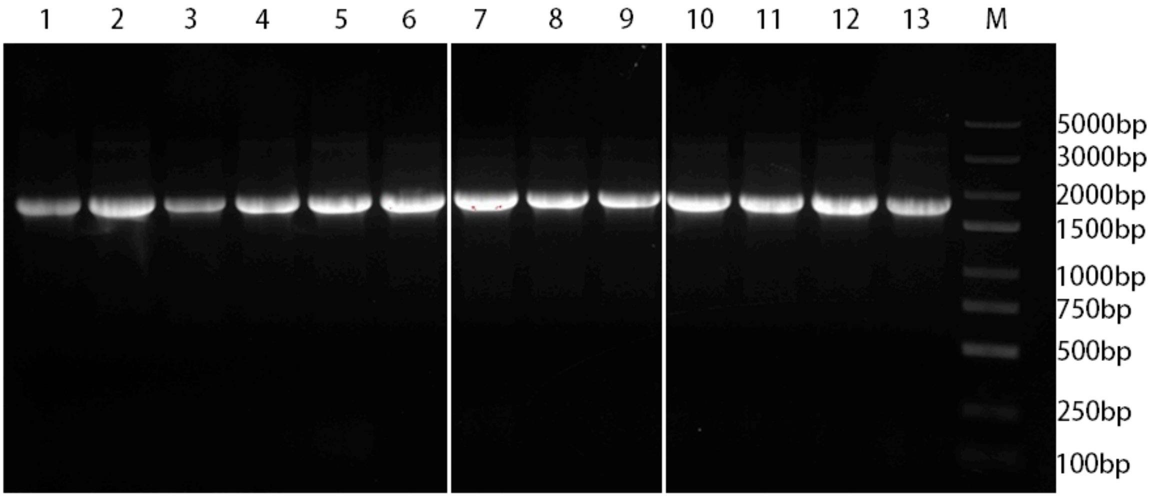

Figure S3. Electrophoresis results of 18S rDNA amplification products in 13 species of the genus *Ipomoea*.
